# Effectiveness of an Attachment-Informed Working Alliance in Interdisciplinary Pain Therapy

**DOI:** 10.3390/jcm8030364

**Published:** 2019-03-14

**Authors:** Ann-Christin Pfeifer, Pamela Meredith, Paul Schröder-Pfeifer, Juan Martin Gomez Penedo, Johannes C. Ehrenthal, Corinna Schroeter, Eva Neubauer, Marcus Schiltenwolf

**Affiliations:** 1 Department of Orthopedics, Trauma Surgery and Paraplegiology, Heidelberg University Hospital, Schlierbacher Landstr. 200a, 69118 Heidelberg, Germany; corinna_schroeter@t-online.de (C.S.); eva.neubauer@med.uni-heidelberg.de (E.N.); marcus.schiltenwolf@med.uni-heidelberg.de (M.S.); 2 Institute of Medical Psychology, Center for Psychosocial Medicine, University Hospital Heidelberg, Bergheimer Str. 20, 69115 Heidelberg, Germany; Johannes.Ehrenthal@med.uni-heidelberg.de; 3 School of Health and Rehabilitation Sciences, The University of Queensland, St. Lucia, QLD 4072, Australia; p.meredith@cqu.edu.au; 4 School of Health, Medical and Applied Sciences, Central Queensland University, North Rockhampton, QLD 4701, Australia; 5 Institute of Psychosocial Prevention at the Center for Psychosocial Medicine, University Hospital Heidelberg, Bergheimer Str. 54, 69115 Heidelberg, Germany; paul.schroeder-pfeifer@med.uni-heidelberg.de; 6 CONICET and Universidad de Buenos Aires, C1053 CABA, Buenos Aires, Argentina; jmgomezpenedo@gmail.com

**Keywords:** chronic pain, attachment theory, attachment-informed intervention

## Abstract

Attachment theory provides a useful framework for understanding individual differences in pain patients, especially with insecure attachment shown to be more prevalent in chronic pain patients compared to the general population. Nevertheless, there is little evidence of attachment-informed treatment approaches for this population. The present study compares outcomes from two different attachment-informed treatment modalities for clinicians, with outcomes from treatment as usual (TAU). In both intervention groups (IG1 and IG2), clinicians received bi-monthly training sessions on attachment. Additionally, clinicians in IG1 had access to the attachment diagnostics of their patients. All treatments lasted for four weeks and included a 6-month follow up. A total of 374 chronic pain patients were recruited to participate in this study (TAU = 159/IG1 = 163/IG2 = 52). Analyses were carried out using multilevel modeling with pain intensity as the outcome variable. Additionally, working alliance was tested as a mediator of treatment efficacy. The study was registered under the trial number DRKS00008715 on the German Clinical Trials Register (DRKS). Findings show that while IG2 was efficient in enhancing treatment outcomes, IG1 did not outperform TAU. In IG2, working alliance was a mediator of outcome. Results of the present study indicate that attachment-informed treatment of chronic pain can enhance existing interdisciplinary pain therapies; however, caveats are discussed.

## 1. Introduction

Chronic pain syndromes are a result of complex interactions between biological, psychological, and social influences, including patients’ beliefs about their self-efficacy, hypervigilant monitoring of bodily sensations, and familial conflict or social support [1,2,3]. Due to maladaptive behavior or cognitive responses to acute episodes of pain stemming from these interactions, the pain may become chronic, affecting the long-term course [4].

Attachment theory provides a useful framework to classify patients’ relatively stable cognitive, emotional, and behavioral response styles to stressors (such as pain). These attachment-related response styles, or attachment patterns, have been linked to disease processes in general [5,6,7,8] and to pain-related diagnoses and processes in particular [9,10]. Hence, the individual attachment pattern can provide some indications regarding how chronically ill patients behave in treatment; for instance, with regards to health-care utilization, trust, and compliance with the treatment [6,7] as well as self-management [11] and coping strategies used [8].

**Figure 1:**
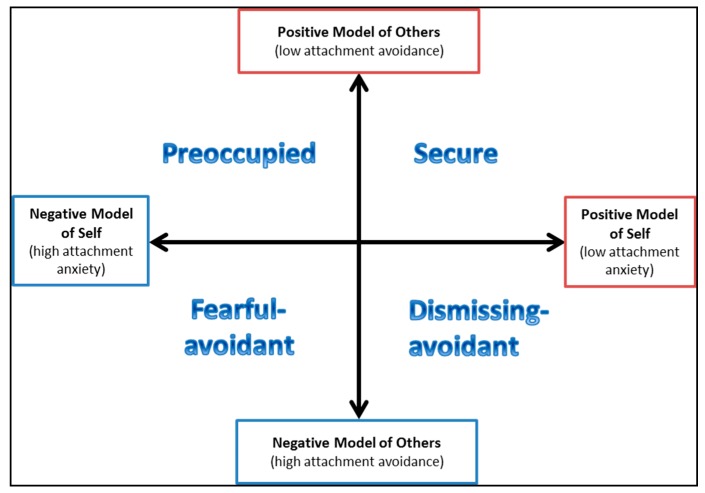
Different attachment styles described by Griffin and Bartholomew [15].

Based on their dominant response patterns, adults can be classified into one of four attachment styles—one secure style, and the three insecure styles: dismissing, preoccupied, and fearful [12,13] (see Figure 1). Attachment can also be operationalized as dimensions, with low scores on both attachment anxiety and avoidance representing secure attachment, and high scores in either attachment anxiety or avoidance (or both) representing insecure attachment [14].

Evidence in the pain literature suggests that these attachment styles relate to patients’ stress response, beliefs about their ability to cope with the experience of pain, perceptions of the pain as threatening, and specific interaction patterns with partners and health care personnel [16,17]. In general, patients with insecure attachment patterns report higher levels of pain [18,19,20], higher burden of disability [21], lower levels of pain self-efficacy [17], less functional and more dysfunctional coping strategies such as catastrophizing [22], and greater levels of depression and anxiety [23,24]. These findings are especially relevant given that insecure attachment styles are overrepresented in patients with chronic pain [20,21,25,26].

Given available evidence, it is likely that insecurely attached patients have different needs in terms of both their relationship with the clinician and the treatment [27]. Examples of attachment-informed therapies can be found within family therapy [28,29], psychoanalytic therapy [30], therapy for personality disorders [31], and within psychotherapy in general [32]. Traditional interdisciplinary pain therapy includes physical activation, improvement of mobility, the ability to relax, occupational therapy, psychological pain management, reduction of pain killer intake, and coping-related interventions. An attachment-informed treatment approach for people in pain may assist clinicians to deepen their understanding of the individual patient, individualize treatment, and develop therapy as a safe place, potentially improving outcomes; however, no evidence in the pain field exists to support this proposition.

In the present study, clinicians in a four-week interdisciplinary multimodal pain treatment program at the Heidelberg Orthopedic Hospital, Heidelberg University Clinic, were trained in: attachment theory, attachment-related individual differences, related clinical implications, and suggestions for building a meaningful working alliance. This training was expected to facilitate the attainment of the program’s aims by enhancing the working alliance, the therapists’ ability to provide a secure base for patients, and the therapists’ understanding and support of their patients’ individual attachment-based motivations and needs. The aims of this study were to examine whether: (a) there is a main effect of group (two attachment-informed groups (IG1 and IG2) versus treatment as usual (TAU)) on treatment outcome; (b) group effect is mediated by working alliance; and (c) working alliance is moderated by insecure attachment. The main hypotheses are that:

(1) Patients in IG1 and IG2, who both receive an attachment-informed multidisciplinary treatment, will report a larger mean reduction in pain intensity between pre-treatment, post-treatment, and follow-up assessments than patients in the TAU group who receive state-of-the-art multidisciplinary treatment.
(2) As the interventions (IG1 and IG2) are specifically designed to improve the working alliance, we expect higher ratings for the working alliance in IG1 and IG2 compared to TAU.
(3) The quality of the working alliance will be the core mechanism of change in IG1 and IG2; that is, it will be the mediating variable between intervention and outcome.
(4) As patients with higher levels of insecure attachment might not profit from the alliance in the same way as securely attached patients, we expect this mediation effect to be moderated by insecure attachment.

## 2. Materials and Methods

### 2.1. Participants

**Figure 2:**
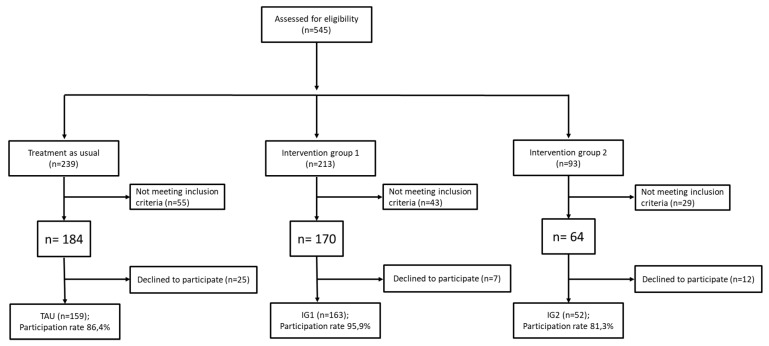
Trial flow chart describing the recruitment process of all three study arms.

Of the 545 patients attending the Heidelberg Orthopedic Hospital, University Clinic Heidelberg, between March 2012 and January 2016, 127 patients did not meet the inclusion criteria to be treated in the clinic (see below) and another 44 patients declined to participate. Therefore, a total of 374 (68.6%) were recruited to this study. As seen in the flow chart in Figure 2, 159 of these participants were assigned to the TAU group, 163 to the IG1 group, and 52 to the IG2 group. Table 1 displays descriptive details for the demographic variables of the patient population.

### 2.2. Inclusion and Exclusion Criteria

All participants were enrolled as day-clinic patients in the orthopedic clinic of the Heidelberg University Hospital, and participated in a four-week outpatient multidisciplinary pain treatment program, including physiotherapy, occupational therapy, music and dance therapy, and individual and group psychotherapy. To attend this clinic they must: (1) have experienced chronic pain for at least six months, for which pain intensity, location, and spreading was not fully explained by specific somatic pathology; (2) be between 18 and 80 years of age; (3) have previously received standard treatment consisting of at least one rehabilitation program or two inpatient treatments, which did not yield lasting effects; and (4) have a diagnosis of somatoform disorder according to DSM-IV. In order to check whether or not these inclusion criteria were fulfilled, comprehensive diagnostic imaging and examination by an orthopedic specialist was conducted, as well as an interview with the structured clinical interview for DSM-IV (SCID) by a trained psychologist.

Exclusion criteria were:

- High C-Reactive Protein (CRP) levels as an indicator of rheumatoid arthritis;
- Acute inflammation of the spine;
- A tumor;
- A diagnosis of psychosis;
- A diagnosis of a bipolar or neurological disorder;
- Insufficient ability to communicate in German.

Use of medication was discouraged throughout the treatment, and the number of patients taking opioids or equivalent drugs in the outpatient clinic was very low (only 8.6%). While information regarding medication usage (including antidepressants and antiepileptics) was gathered at all time-points, it was not part of the exclusion criteria.

### 2.3. Design

All study procedures were approved by the Institutional Ethics Review Board of the Medical Faculty, University of Heidelberg. All procedures were in accord with the newest version of the Declaration of Helsinki [33], as well as with the guidelines for good clinical practice.

**Figure 3:**
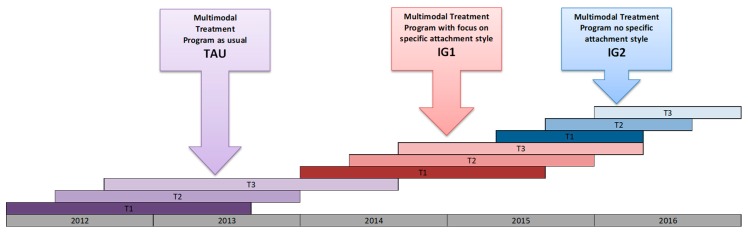
Study design.

After a briefing about the study procedures and aims, all participating patients provided written consent. The study was conducted in a block design with three patient groups (TAU, IG1, and IG2) and three assessments times (before treatment = T1, post-treatment = T2, and 6-months follow-up=T3; see Figure 3). Patients who were registered between March 2012 and September 2013 were assigned to the TAU group, patients who were registered between March 2014 and June 2015 were assigned to IG1, and patients who were registered between June 2015 and January 2016 were assigned to IG2. All measures were given in paper pencil format and completed in the clinic for T1 and T2. For T3, the questionnaires were mailed to the participating patients. A randomized controlled trial was not suitable for this study because of ethical concerns that patients would be put on a waiting list for several months. A block design increases the chance that the key influence on outcomes is the intervention used, and was approved by the Institutional Review Board.

After data collection for the TAU group was complete for all time points, the health care personnel of the outpatient pain clinic received two initial 90-min training sessions on attachment theory and its use in the therapeutic context. The intervention training offered to the healthcare professionals working at the outpatient pain clinic included both (a) general directions for building a meaningful working alliance; and (b) guidelines for the clinicians to enable them to tailor treatment to the specific needs of individual patient attachment styles and behaviors. More attachment-related training sessions, alternating with supervision meetings, were held on a monthly basis to assist clinicians in the practical application of this approach during interventions for the second (IG1) and third (IG2) study samples. Further, “situations” (e.g., instances in which the patient misses entire therapeutic sessions or appears too late to them on a regular basis), which are perceived to be critical for forming a working alliance, were discussed at the weekly meetings. These situations were subsequently used to structure case discussions in the bi-monthly 90-min training sessions.

The only difference between the IG1 and IG2 interventions was that, in IG1 only, weekly team meetings incorporated case reviews with discussion regarding the attachment diagnostics (i.e., individual attachment styles) of each patient. The IG2 group also had weekly team meetings, but the clinicians were not informed about the specific attachment style of each patient. Instead, it was expected that after receiving the attachment-based training sessions, the team would be more sensitive to the individual attachment behavior of patients without knowing the specific attachment style.

### 2.4. Interdisciplinary Multimodal Pain Treatment

The interdisciplinary multimodal pain therapy provided to the patients in the TAU group consisted of an intensive, structured interdisciplinary program provided in an outpatient setting with five hours of treatment per day, five days per week, for four weeks. The treatment included physiotherapy, occupational therapy, psychotherapy, and medical treatments in both individual and group modalities. Additionally, patients could participate in Nordic walking and dance and music therapy, as well as relaxation training and guided physical activity supervised by physiotherapists.

### 2.5. Attachment-Informed Training

In the attachment-informed approach, the same clinicians received training about attachment theory and attachment-informed treatment principles [34]. The primary aim was to improve the working alliance by improving the therapists’ ability to: (a) provide a secure base for patients; and (b) understand and deal with patients’ individual attachment-focused motivations and needs. They then sought to integrate these attachment approaches and techniques within the treatment as usual approach. The usual aims of the multimodal pain treatment were retained. Importantly, the approach did *not* aim to modify underlying insecure attachment patterns, which would have been unrealistic within four weeks in this setting.

The motive-orientated working alliance (former known as complementary therapeutic relationship) [35] informed the development of general guidelines for an improved working alliance. This approach emphasizes the underlying motives (such as attachment motives) of patients. Using existing literature on the application of attachment ideas to specific therapeutic settings (e.g., borderline personality disorder, depression, medically unexplained symptoms, and family and couple therapy [30,36,37,38]) as a starting point, specific guidelines were created for developing a working alliance for each attachment style. As an example, patients with anxious attachment styles might benefit more from an initially concordant approach that emphasizes the therapist’s role as a secure base. These patients might feel overwhelmed by a program which is too quick to emphasize autonomy, possibly reinforcing existing fears of rejection and abandonment. On the other hand, avoidantly attached patients might feel uncomfortable with high levels of proximity or intimacy, and the amount of guidance and care favored by anxiously attached patients [39,40].

In the interdisciplinary setting, it was necessary that the attachment-based approach be readily employed by healthcare professionals with diverse professional backgrounds (e.g., doctors, physiotherapists, occupational therapists, and music and dance therapists); therefore, all guidelines needed to be easily incorporated into all professional approaches.

### 2.6. Outcome Measures

#### 2.6.1. Pain Visual Analogue Scale (VAS)

Current pain was assessed by a VAS, asking the patients to rate their acute pain during the present day on a scale ranging from 0 to 100. Similarly, average pain over the previous week was assessed using a visual analogue scale ranging from 0 to 100. For rating purposes these scales were collapsed to indicate values between 0 and 10. Visual analogue scales have been proven to provide a valid and reliable way of measuring chronic and acute pain [41,42]. VAS were assessed at each time point.

#### 2.6.2. Oswestry Low Back Pain Disability Questionnaire [43]

The Oswestry Low Back Pain Disability Questionnaire is a self-report measure of the functional disability of the patients and consists of 10 items assessing pain and disability in specific contexts of life to measure functional disability due to pain [44]. The items are scored on a 6-point Likert scale ranging from “no functional disability” (0) to “complete functional disability” (5). The original measure is considered the gold standard in assessing functional disability due to back pain [45]. The present study used the German version of this questionnaire, which has shown very good internal consistency (α = 0.94) [43]. The original questionnaire (in English) has also shown good construct validity and test-retest reliability over a span of two weeks (r = 0.82; [46]). In the present study, the Oswestry Low Back Pain Disability Questionnaire showed good internal consistency, with Cronbach’s α values ranging from 0.80 at T1 to 0.88 at T3. The Oswestry Low Back Pain Disability Questionnaire was assessed at each time point.

#### 2.6.3. Experiences in Close Relationships Scale Revised 12—German Version (ECR-RD12) [47]

The ECR-RD12 is a German short version of the ECR-RD scale, which has previously revealed very good internal consistency (α = 0.91–0.92; [48]). The ECR-RD12 is a self-report measure of attachment, with questions referring to participants’ behavior in romantic relationships. The ECR-RD12 consists of 12 items, with 6 items loading on two scales: avoidant attachment and anxious attachment. Items are scored on a 7-point Likert scale ranging from “disagree strongly” (1) to “agree strongly” (7) [47]. The original English instrument has a stable factor structure, as well as good test-retest reliability (r = 0.80–0.83) and construct validity [49]. The attachment patterns measured by the ECR-RD12 were treated as continuous variables with mean values computed. Additionally, attachment insecurity was derived from the ECR-RD12 as a sum score of both scales, with high values representing attachment insecurity and low scores representing attachment security. In the present study, the ECR-RD12 showed good internal consistency, with Cronbach’s α values of 0.78 for anxious attachment and 0.82 for avoidant attachment. The ECR-RD12 was assessed at T1 only.

#### 2.6.4. Inpatient and Day-Clinic Experience Scale—German Version (German TSEB/English IDES) [50]

The TSEB is a self-report questionnaire with 35 items, which assesses various facets of the working alliance specifically designed for day-clinic patients [50]. Seven scales are calculated: bond with individual therapist, bond with therapeutic team, agreement on tasks and goals, cohesion with the patient group, self-disclosure, critical attitude, and positive self-view. Items are scored on a 6-point Likert scale ranging from “not at all true” (1) to “completely true” (6). The authors have reported mixed internal consistency, ranging from α = 0.53 for critical attitude to α = 0.89 for positive self-view, while they found evidence of construct validity with good confirmatory factor analysis model fit. In the present study, the TSEB showed varying internal consistency ranging from poor to high (from α = 0.58 for critical attitude to α = 0.89 for bond with individual therapist), congruent with the results of the validation study. The bond with therapeutic team subscale was primarily used, as this was deemed best fitting for the day-clinic setting. The TSEB was assessed at T2.

### 2.7. Statistical Analyses

SPSS 22 [51] was used for descriptive analysis and data management, while R [52] was used for missing data analysis and handling of outliers. Power was computed analytically via the R package “powerlmm”. Assuming a small to medium effect of the treatment of Cohens *d* = 0.5, a power analysis was computed for an ICC of 0.2 for three time-points. According to the power analysis, 95% power was achieved at a group size of *n* = 160. Unfortunately, due to the nature of the block design and complications in recruitment, IG2 had only *n* = 52 willing participants. Thus, IG2 was underpowered at only 51% power.

The data contained 9.82% missing values. The group of complete cases did not differ from the group with missing values on one or more variables in mean or standard deviation on any variable of interest. All analyses were conducted assuming the data was missing at random (MAR). Under MAR, observation missingness is assumed to be unrelated to the dependent variable at dropout [53]. Multiple imputations by chained equations (MICE) [54] with 20 iterations were used to impute missing values for available time points. MICE produces asymptotically unbiased estimations of the data under MAR assumptions [55]. Using *p* > 0.001 for the χ^2^ value of the Mahalanobis distance as a measure of multivariate outliers, no outliers were identified.

HLM7 software was used for multilevel modeling [56]. We used longitudinal multilevel models with measurements over time (level 1) nested in patients (level 2), since it can be expected that measurements within patients over time are non-independent [57]. Multilevel models offer a good way of handling unbalanced designs, accounting naturally for the different number of measurements per person [58,59]. To answer the questions regarding whether or not there were significant differences in level of the outcome variable (pain intensity) at six months follow-up and weekly rate of change during treatment and follow-up period, dependent on treatment group (IG1 vs. IG2 vs. TAU) we tested several models. For each outcome variable, we tested a two-level conditional model with time in weeks (centered at the end of the 6-month follow-up) as the only level-1 predictor. At level two, we included the treatment conditions as well as attachment anxiety as predictors both of the intercept and the slope of the model. As patients were nested with therapists, but only four therapists participated in the study, instead of conducting three-levels to control for therapist effects, we decided to include the therapist as a covariate (dummy coded) in all models.

Since there was prior evidence from another study conducted at the Orthopedic Hospital that patient trajectories would be markedly different during treatment as opposed to during follow up, we decided to use a piecewise modeling approach. In the earlier study, symptoms declined steeply during treatment, and started increasing again during follow up. Change in each piece of the model was estimated using the technique outlined by Smith and colleagues [60] by providing the estimated error variance at level 1 for the outcomes in the model [60,61]. The level-1 error variance of each outcome measure was estimated as the product of its measurement error (1-Cronbach’s α) and the variance of the measure at each time-point.

Full maximum likelihood was used as the estimator in all models. Significance values and standard errors for fixed effects were computed using Kenward-Roger approximation [62]. Plotting the fitted against the residual values did not indicate non-constant error variance for any of the models and visual inspections of Q-Q plots did not reveal marked non-normality for any of the models.

To test mediation and moderated mediation effects, we used PROCESS macro version 2.11 for SPSS version 22.0 [63]. For these models, the Empirical Bayes estimates of patient’s scores at the end of follow-up and of the weekly rated of change, estimated in the above-mentioned two-level models, were used as the outcome variables [57]. Hayes’s models 1 and 14 were used to test mediation and moderated mediation effects.

## 3. Results

In terms of descriptive statistics, there were no significant differences between the three groups in terms of the core study variables (see Table 2).

Table 3 summarizes correlations among core study variables at intake using Pearson’s correlation for continuous variables and Spearman-rank coefficients for non-continuous variables. Alpha levels were adjusted using Bonferroni correction.

### 3.1. Treatment’s Main Effects Analysis

**Figure 4:**
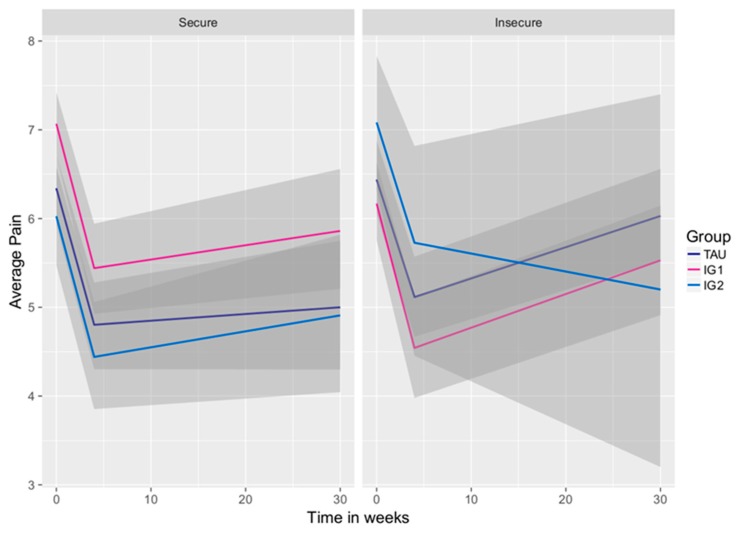
Effect of attachment on average pain across treatment groups.

Concerning Hypothesis 1, the conditional model including average pain as the outcome variable showed no difference between either IG1 or IG2 to TAU at post-treatment (see Figure 4) but a significant difference at 6-month follow-up in average pain for IG1 compared to IG2 (γ01 = −0.92, SE = 0.45, t(358) = −2.027, *p* < 0.05), in favor of IG2 (see Table 4). Additionally, IG2 worked markedly better in the long run for patients with high attachment anxiety (see Figure 4). While having significantly worse pain intensity scores in IG2 after treatment compared to TAU and IG1 (γ03 = −0.52, SE = 0.22, t(358) = 2.32, *p* < 0.05), patients with high attachment anxiety achieved the lowest scores of pain at follow-up (γ23 = −0.02, SE = 0.01, t(358) = −1.78, *p* = 0.08), although this effect did not reach significance.

Figure 4 shows the effect of insecure attachment across the three treatment groups over time. Gray shaded areas indicate 95% confidence intervals around the parameter estimates, with darker areas indicating overlapping confidence intervals.

### 3.2. Mediational Effects Analysis

Although there was no significant difference between TAU and the two intervention conditions, mediational analyses were conducted to see if there was an indirect effect of treatment condition on outcome by working alliance, as specified in Hypotheses 2 and 3. For these models we used a dummy variable as the independent variable comparing IG2 with TAU (i.e., IG2 = 1, TAU = 0). IG2 was compared to TAU, since previous analysis hinted that these groups provided the greatest potential to explore this mediation effect by way of being conceptually different and also boasting bigger outcome differences than IG1 vs. TAU. The working alliance with team score provided by the TSEB was introduced as the mediator. For outcome variables we used the estimated score at post-treatment and at follow-up, as well as the slope (weekly rate of change) of pain intensity, leading to a total of six mediation analyses.

Regardless of the model, the treatment condition was significantly related to scores at TSEB (B = 0.23, SE = 0.12, t(179) = 2.003, *p* < 0.01). Patients in IG2 revealed a TSEB score of an estimated 0.23 units higher than patients in the control group. Furthermore, TSEB scores were significantly related with average pain at follow-up (B = −0.21, SE = 0.10, t(178) = −2.183, *p* = 0.03) and the weekly rate of change in average pain (B = −0.002, SE = 0.001, t(178) = −2.227, *p* = 0.03). Overall, the indirect effect of treatment condition by TSEB scores was significant for average pain at the end of follow-up, but not for the weekly rate of change in average pain.

In the mediational models for current pain, we again found that TSEB scores were significantly related with current pain at the end of follow-up (B = −0.27, SE = 0.14, t(178) = −1.986, *p* < 0.05) and the weekly rate of change in current pain (B = −0.002, SE = 0.001, t(178) = −2.075, *p* = 0.04). There was a significant indirect effect of treatment by TSEB scores on current pain at the end of follow-up; however, the indirect effect of treatment by TSEB scores on weekly change in current pain was not significant. On the other hand, there was no significant direct effect of treatment on current pain at follow-up (B = −0.13, SE = 0.21, t(178) = −0.616, *p* = 0.54) or in current pain weekly change (B = −0.001, SE = 0.001, t(178) = −0.596, *p* = 0.55).

### 3.3. Moderated Mediational Effects Analysis

**Figure 5:**
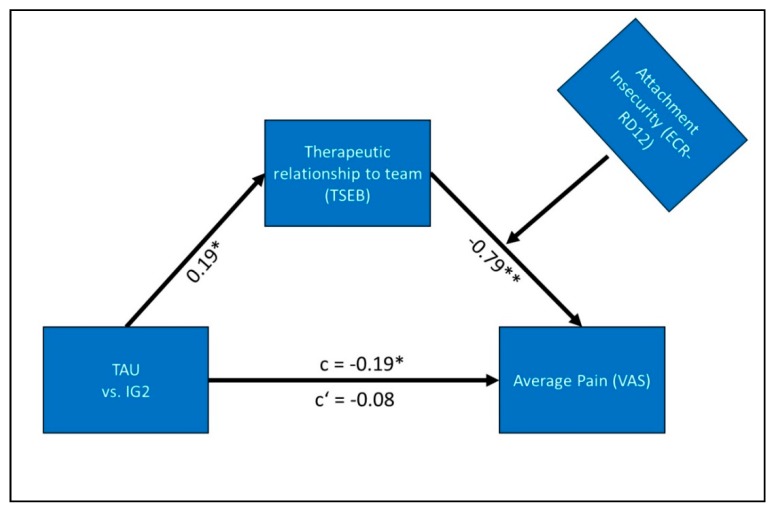
Summary of results for moderated mediation analysis. Note: *p* < 0.05 = *, *p* < 0.01 = **, c = direct effect before mediation, c‘ = direct effect after mediation.

We conducted moderated mediational effects analysis to check if the mediational effects reported (indirect effect of treatment by TSEB scores) were, in turn, moderated by patient attachment pattern (Hypothesis 4). As presented in Figure 5, attachment anxiety significantly moderated the mediational effect of treatment by TSEB scores on average pain at the end of follow-up (B = 0.23, SE = 0.09, t(155) = 2.628, *p* < 0.01) and the weekly change in average pain (B= 0.003, SE = 0.001, t(155) = 2.650, *p* < 0.01).

**Figure 6:**
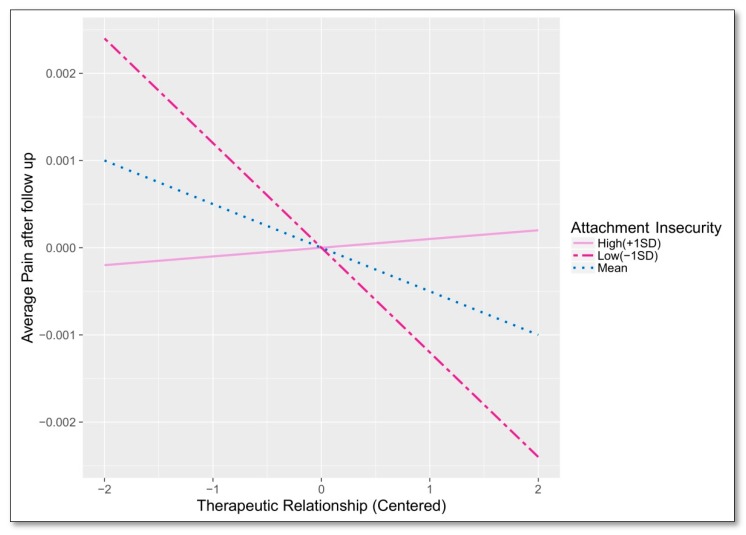
Moderated effect of attachment insecurity on the relationship between working alliance and average pain after follow up.

In summary, the mediation analysis indicated a difference in pain reduction between IG2 and TAU that is mediated by working alliance, measured by the TSEB. This effect, in turn, is dependent on attachment insecurity, as shown in Figure 6. High values of attachment insecurity negate the positive effect of the working alliance, while low values reinforce it. This moderating effect extends to both the average level of pain at follow-up (−1 SD ECR-RD12 Insecurity B = −0.11, SE = 0.7; +1 SD ECR-RD12 Insecurity B = 0.01, SE = 0.03), and rate of change during therapy (−1 SD ECR-RD12 Insecurity B = −0.0012, SE = 0.0008; +1 SD ECR Insecurity B = 0.0001, SE = 0.0003).

## 4. Discussion

The primary aim of this study was to examine whether providing attachment-informed training to clinicians in an interdisciplinary pain program could influence pain outcomes compared to treatment as usual (TAU). According to Hypothesis 1, it was anticipated that this would be the case. Results only partially supported this hypothesis, however. Patients in IG2 reported a larger mean reduction in average pain intensity between pre-treatment and post-treatment assessments compared to patients in both IG1 and TAU groups. Perhaps surprisingly, IG2, in which therapists were *not* informed of patient attachment style, outperformed IG1, where therapists knew the attachment styles. Thus, this additional knowledge seemed to have had an adverse effect. There are a number of possible explanations for this phenomenon. Most likely, with therapists at the IG1 stage being new to attachment theory, they will have been consolidating information and gaining new perspectives throughout the IG1 stage, which would presumably support practice during IG2. The clinicians in IG1 may have felt overwhelmed, having access to a large amount of new information and to the patients’ attachment style, and trying to integrate these components “on the job”. The changed role of the therapist, in which they serve as a form of substitute attachment figure for the patients that has to attune to each individual attachment pattern by addressing the specifically related needs [64], also takes time to develop. These considerations are particularly relevant given the short length of the treatment (four weeks). It may also be that knowing a patient’s attachment style might evoke a form of unconscious stigma on the part of the therapist, which may impact on the therapeutic relationship. Another possible explanation is that attachment, as measured by the ECR-RD12, is not representative of the attachment behavior exhibited during therapy, therefore misleading clinicians in IG1. Finally, the smaller number of participants in IG2, and reduced power, may have impacted on results. Further research is needed to gain clarity about these possible explanations.

The interdisciplinary pain therapy includes physical activation, improvement of mobility, the ability to relax, occupational therapy, psychological pain management, reduction of pain killer intake, and coping-related interventions. An ordinary treatment can last up to 4 weeks for a full-time intensive outpatient treatment. Due to the limited time available, it is generally very difficult to build a stable and trusting work-alliance between the therapist and the patient to allow the patient to properly take in the content of the therapy. Even though it is much harder for insecure patients to establish and maintain a stable and trusting working alliance [21,65], the development of trust is essential for the success of therapy [40,66]. One therapeutic approach that already includes these relationship related aspects is the psychodynamic interactional group therapy by Nickel and Egle [67] that already works with these relationship aspects in a clinical setting, with a focus on the working alliance and conflict management during 40 sessions.

The second hypothesis was that the intervention groups (IG1 and IG2) would produce stronger average working alliances compared to TAU. This was partly supported, with patients in IG2 reporting significantly better working alliances compared to those in TAU. Working alliances for patients in IG1 did not differ from those in TAU.

The third hypothesis suggested that the quality of the working alliance would be the core mechanism of change in IG1 and IG2 (i.e., the mediating variable between intervention and outcome). As expected, working alliance was found to be a strong mediator between the intervention effect and treatment outcome, suggesting that training staff in attachment theory and its implications for people in pain can help to improve the working alliance, and therefore strengthen outcomes. This is consistent with expectations based on parent-infant attachment-based interventions, where training in attachment theory enhanced maternal-sensitivity and infant-security [68]. Literature and some empirical evidence point to the importance of the working alliance for the course of the treatment and its outcomes, as well as for the maintenance of positive treatment outcomes after therapy ends [69,70]. The results of this study correspond to attachment theoretical assumptions [7,71] as well as to the impact of the working alliance [72]. This is one of first studies to consider these assumptions in a clinical setting with a longitudinal design, and the first to do so with chronic pain samples.

As expected in Hypothesis 4, the link between working alliance and pain outcome was moderated by insecure attachment. Patients with higher levels of insecure attachment reported poorer working alliances compared to securely attached patients, with implications for pain outcome. This finding was evident despite attachment-informed intervention provided in this study, suggesting that this intervention did not counter the effects of attachment insecurity on pain outcome. While anticipated, based on previous research, this finding suggests the need for attachment-informed modifications to treatment that extends beyond the therapeutic alliance. The mentalization-based approach [73] has been successfully utilized with mostly insecure attached patient groups before (e.g., [74,75]) and could provide a useful addition in working with chronic pain patients. In contrast to the focus of present study on how to establish a good therapeutic alliance with insecure patients, the mentalization-based approach could aid in understanding how the communication in these therapeutic alliances works and how the patient mentalizes the relationship. This might help explain why the insecure patients were not able to profit from the therapeutic alliance in the same way as did secure patients.

Findings support working alliance as a mechanism of change linked to patient attachment. Nevertheless, the path model also indicated that the insecure patients in IG2 were the only insecure patients who did not experience deteriorations in pain during the post-treatment phase. Although the mean difference in pain at the 30-week follow-up was non-significant, this trend hints at the attachment-specific training having a positive effect on post-treatment adjustment to pain [76,77]. The results from TAU, on the other hand, replicate the evidence from the empirical literature stating that insecurely attached patients have, on average, poorer treatment compliance [7,71] and adjustment to pain [4,9,78].

### Limitations

The primary limitation for this study can be found in the study design. The block design was chosen even though the optimal design for the study would have been a randomized controlled trial (RCT), with patients being randomly assigned to one of the treatment arms with separate groups of treating clinicians. However, this was not feasible in this orthopedic hospital setting. The very limited number of clinicians working at the outpatient department would have made it impossible to divide the clinicians into more than one interdisciplinary pain treatment at a time. A future study replicating the results of the current study might use a multicenter trial in order to control for spillover effects, while providing an adequate number of clinicians for an RCT design.

Another limitation is the failure to control for therapist adherence to the treatment guidelines. While the clinicians were regularly asked during the weekly team meeting whether or not they implemented the training contents into their treatment routine, no adherence data is available. Future studies might profit from development of an intervention manual with clearly defined treatment characteristics and working mechanisms to support development of systematic adherence ratings. These might then be ascertained either from expert rated videos of therapy sessions, or a comparable approach, such as a manualized adherence rating. This information could be used as a control variable or descriptively to support interpretation of findings.

While the main objective of the present study was to compare the three treatments, i.e., investigating between-person effects, we were also interested in the trajectories of treatment over time in our treatment groups. The short duration of the treatment combined with relatively long assessments at each measurement point has resulted in having only the minimum number of time points needed for longitudinal modeling. This, combined with large standard errors for our estimates of within-person effects, render low levels of certainty in those estimates. For future studies of multimodal pain therapies with attachment focus, a shorter assessment battery with more time points is needed to properly investigate the trajectories in patients’ symptoms over time.

A further methodological issue is the sample size of IG2 and the high dropout rate at follow-up across all treatment arms. While the non-significant *t*-tests across dropouts and non-dropouts suggest that dropout is not systematically related to outcome, the question remains why the dropout at T3 was so high. Although significant effects between treatments were found, the results of the present study need to be replicated in a future study with all treatment arms being powered equally.

Finally, the intervention was provided over only a four-week period. If adapted for longer outpatient settings, more pronounced differences may be seen over time as, hypothetically, attachment patterns might slowly alter over the course of therapy, increasing the positive effect of the working alliance on pain over time.

## 5. Conclusions

The results of the present study provide preliminary support for the utility of incorporating attachment-informed interventions with existing multimodal pain therapies in short-term outpatient settings. Although the clinicians trained in the attachment-informed treatment only had four weeks to implement the treatment, this approach was more effective in reducing perceived pain intensity in IG2 relative to TAU. Findings suggest that one reason for this was the facilitation of a more stable working alliance between the therapist and the patient in the attachment-informed treatment. Findings also suggest that classifying patients into one of the four attachment categories prior to treatment may not be needed to build a stable working alliance. As a result of this study, a number of needs are identified. First, there is a need for a written manual with a detailed description of the intervention to support clinicians to adhere to and integrate the new techniques of the intervention into their daily treatment routine. Second, based on this manual, measures of treatment adherence by clinicians should be developed. Finally, more in-depth attachment-informed treatments should be developed, manualized, and evaluated. It is anticipated that these steps will contribute to even greater and more lasting clinical improvements, especially for those with insecure attachment patterns.

## Figures and Tables

**Table 1:** Descriptive details for demographic variables for the patient population and comparisons across treatment groups, *n* = 374.

Variable		TAU *n* = 159		IG1 *n* = 163		IG2 *n* = 52		Statistical Test	*p* Value
		*M*/%	*SD*	*M*/%	*SD*	*M*/%	*SD*		
Age		66.67	12.04	58.90	13.01	67.31	12.90	*F*_(2,367)_ = 0.81	0.45
Gender	Female	54%	-	54%	-	52%	-	*χ*^2^_(2)_ = 2.50	0.29
Marital status	Married	61%	-	58%	-	58%	-	*χ*^2^_(2)_ = 0.32	0.85
	Divorced	18%	-	15%	-	21%	-	*χ*^2^_(2)_ = 0.99	0.61
	Single	15%	-	21%	-	19%	-	*χ*^2^_(2)_ = 1.84	0.40
	Widowed	6%	-	6%	-	2%	-	*χ*^2^_(2)_ = 1.49	0.47
Employment	Currently working	47%	-	45%	-	67%	-	*χ*^2^_(2)_ = 9.88	0.01*
	Unemployed	53%	-	55%	-	33%	-	*χ*^2^_(2)_ = 9.88	0.01*
	Old-age pension	71%	-	70%	-	58%	-	*χ*^2^_(2)_ = 3.46	0.17
	Disability pension	15%	-	18%	-	27%	-	*χ*^2^_(2)_ = 370	0.16
Education	Lower/middle secondary	81%	-	86%	-	77%	-	*χ*^2^_(2)_ = 2.34	0.27
	College/university	19%	-	14%	-	23%	-	*χ*^2^_(2)_ = 2.34	0.27

Note: TAU = Treatment as usual, IG1 = Intervention group 1, IG2 = Intervention group 2, M = Mean, SD = Standard deviation, * *p* ≤ 0.05.

**Table 2:** Descriptive details of the core study variables and differences between the three treatment groups, *n* = 374.

Variable	TAU		IG1		IG2		Statistical Test	*p* Value
	*M*/%	*SD*	*M*/%	*SD*	*M*/%	*SD*		
Age	53.56	12.04	54.45	13.01	51.92	12.90	*F*_(2,367)_ = 0.81	0.45
Female	66.7%	-	58.9%	-	67.31	-	*χ^2^*_(2)_ = 2.50	0.29
Average Pain	6.44	1.80	6.74	1.79	6.29	1.75	*F*_(2,371)_ = 1.80	0.17
Current Pain	5.97	2.08	6.04	2.26	5.38	2.22	*F*_(2,364)_ = 1.83	0.16
ECR-RD12 Anxiety	2.30	1.39	2.40	1.39	2.37	1.38	*F*_(2,338)_ = 0.21	0.81
ECR-RD12 Avoidance	2.46	1.22	2.54	1.17	2.43	1.19	*F*_(2,340)_ = 0.22	0.81

Note: ECR-RD12 Anxiety= Anxious attachment subscale of the Experiences in Close Relationships Scale Revised 12—German Version, ECR-RD12 Avoidance= Avoidant attachment subscale of the Experiences in Close Relationships Scale Revised 12—German Version.

**Table 3:** Correlations among core study variables, *n* = 374.

I	Gender	Average Pain	Current Pain	Physical Functioning	ECR-RD12 Anxiety	ECR-RD12 Avoidance
Age	0.12 *	0.15 **	0.12 *	0.25 ***	−0.06	0.19 ***
Gender		0.05	0.06	0.09	0.01	0.08
Average Pain			0.70 ***	0.42 ***	0.01	0.02
Current Pain				0.44 ***	0	0.05
Physical Functioning					0.05	0.1
ECR-RD12 Anxiety						0.22 ***

Note: * *p* ≤ 0.05; ** *p* ≤ 0.01; *** *p* ≤ 0.001.

**Table 4:** Results of multilevel model with average pain intensity as outcome, *n* = 374.

Fixed Effect	Coefficient	Standard Error	*t*-Ratio	Approx. *df*	*p*
For Intercept, *β*_0_					
Intercept	4.823	0.116	41.556	358	<0.001
IG1 vs. IG2	−0.923	0.269	−2.11	358	0.058
ECR-RD12 Anxiety	−0.132	0.092	−1.429	358	0.154
IG2 x ECR-RD12 Anxiety	0.522	0.224	2.322	358	0.021
For Piece 1 slope, *β*_1_					
Intercept	−0.364	0.026	−13.529	358	<0.001
IG1 vs. IG2	0.028	0.073	0.396	358	0.693
ECR-RD12 Anxiety	0.013	0.020	0.660	358	0.510
IG2 x ECR-RD12 Anxiety	−0.052	0.064	−0.812	358	0.417
For Piece 2 slope, *β*_2_					
Intercept	0.018	0.005	3.332	358	<0.001
IG1 vs. IG2	−0.024	0.016	−1.498	358	0.135
ECR-RD12 Anxiety	0.001	0.004	0.023	358	0.981
IG2 x ECR-RD12 Anxiety	−0.021	0.011	−1.774	358	0.077

Note: Approx. df = Approximate degrees of freedom, * *p* ≤ 0.05; ** *p* ≤ 0.01; *** *p* ≤ 0.001.
